# Relationship between obstetric history and recurrent urinary infections

**DOI:** 10.1038/s41598-021-98116-3

**Published:** 2021-09-20

**Authors:** Cynthia Vanaclocha-Ferrer, Barbara-Yolanda Padilla-Fernandez, Magaly-Teresa Marquez-Sanchez, María-Helena Garcia-Sanchez, María-de-la-O Rodriguez-Martin, Nayra Hernandez-Navarro, Cristina Domenech-Perez, Lauro-Sebastián Valverde-Martinez, María-Carmen Flores-Fraile, Misericordia Martínez Huélamo, José-Angel Nieto-Barbero, José-Antonio Miron-Canelo, María-Begoña Garcia-Cenador, María-Fernanda Lorenzo-Gomez

**Affiliations:** 1 Department of Surgery, University of Salamanca, 37007 Salamanca, Spain; 2 Urology Section of the Department of Surgery, University of La Laguna, 38200 San Cristóbal de La Laguna, Santa Cruz de Tenerife Spain; 3 Renal Urological Multidisciplinary Research Group (GRUMUR), Biosanitary Research Institute of Salamanca (IBSAL), P.º de San Vicente, 58-182, 37007 Salamanca, Salamanca Spain; 4 Department of Biomedical and Diagnostic Sciences, University of Salamanca, 37007 Salamanca, Spain; 5 Urology Section of the Department of Surgery, University of Valencia, 46010 Valencia, Spain; 6 Department of Urology, University Hospital of Salamanca, 37007 Salamanca, Spain

**Keywords:** Urology, Bladder, Ureter, Urethra, Urogenital diseases, Urological manifestations

## Abstract

Urinary tract infections affect more than 50% of women. 25% derive from recurrent UTI (RUTI). It is not known the relationship between obstetric history and RUTI occurrence. Investigate the relationship between obstetric events and RUTI. Multicenter observational retrospective study. Groups: G.RUTI (n = 294): women with RUTI; G.NON.RUTI (n = 126): women without RUTI (treated and cured of renal cancer). Descriptive statistics, ANOVA analysis of variance (with Scheffe’s test for normal samples and Kruskal–Wallis for other distributions), Fisher's exact test, Pearson and Spearman correlation studies, and multivariate analysis multiple regression were used. Mean age 61.04 years (19–92), G.RUTI: 56.77 years SD 4.46 (19–85). G.NON.RUTI: 71 years SD 6.73 (25–92) (*p* = 0.0001). Obstetric history: Nulliparous G.RUTI: 20 (3.4%) G.NON.RUTI: 90 (71.42%) *p* 0.0001; Eutocic G.RUTI: 416 (70.74%) G.NON.RUTI: 30 (23.8%) *p* 0.0001. Dystocic G.RUTI: 58 (9.86%) G.NON.RUTI: 56 (44.44%) *p* 0.0001. G.RUTI abortion: 102 (17.34%) G.NON.RUTI: 30 (23.8%) *p* 0.1381. Hysterectomy without adnexectomy G.RUTI: 100 (17%) G.NON.RUTI: 18 (14.28%) *p* 0.5640. Hysterectomy with adnexectomy G.RUTI: 100 (17%) G.NON.RUTI: 66 (52.28%) *p* 0.0001. Nulliparity, dystocic delivery, and hysterectomy with adnexectomy are more frequent in women without RUTI, while eutocic births are more associated with RUTI. The most prevalent gynaecological-obstetric history in women with RUTI is eutocic delivery associated with a good health state.

## Introduction

Urinary tract infection (UTI) is the second most frequent human infection after respiratory. For women, risk of having UTI throughout their lives is greater than 50%^1^.

Around 20% of women who suffer a first UTI will present successive UTIs^2^, meeting recurrent UTI criteria, that is, more than 3 per year or more than 2 UTIs in 6 months^3^, in addition, with each episode of UTI, the risk of recurrence increases^4,5^.

They have been described as predisposing factors to suffer RUTI the age, sexual habits, and urinary tract physiological and anatomical conditions. In the age range between 15 and 50 years, main factors are intercourse, diaphragm and/or spermicide use, previous antibiotic therapy, mother with repeated infections, UTI history in childhood and no secretory phenotype of blood cells. Predisposing factors between the ages of 50 and 70 include estrogen depletion, urogenital surgery, urinary incontinence, cystocele, post-void residue, non-secretory status, and a previous UTI history. From age 70, urinary incontinence, permanent catheter, urogenital surgery, mental state deterioration and antimicrobial treatment are the most frequent predisposing factors^6^.

UTI has been associated with urinary incontinence (UI). There are studies that demonstrate the relationship between UI and transient UTI^7^. However, although UTI can associate transient UTI for the duration of the infectious-inflammatory picture, it cannot be confirmed with the data currently available that UTI causes permanent UI^8^.

Renal cancer is the abnormal transformation of renal parenchyma cells, which proliferate abnormally and uncontrollably, losing differentiation and acquiring the ability to spread to adjacent tissues and migrate to other organs^9^.

A higher recurrence has been described in younger patients and a better prognosis of clear cell tumors in the female sex^10^. Functional status is the most important independent prognostic variable, especially in metastatic disease, in which it is related to survival and response to immunotherapy^11^. The presence of symptoms at diagnosis is also a poor prognostic factor, especially weight loss of more than 10% in the 6 months prior to diagnosis. Anatomical factors are included in the pTNM classification system^12–14^.

Parity is the number of vaginal deliveries a woman has experienced, according to The International Federation of Gynecology and Obstetrics (FIGO) in the Guide for First Trimester Screening and Prevention, defined the advance maternal age as ≥ 35 years by the moment of delivery and associated the increase in 4% of urinary infections when the maternal age is more than 40 years. However in nulliparous women, the increased risk of developing any complications as preeclampsia or urinary infections has been reported^15,16^.

Thus, a distinction can be made between nulliparous women who have not undergone any delivery, primiparous women who are in their first vaginal delivery or multiparous women who have undergone one or more deliveries^17^.

Pregnancy and childbirth, two stages in a fertile woman’s life that negatively influence pelvic floor’s statics and functionality balance. Although biological mechanisms of its injury have not been completely established, compression, stretching and tearing, nervous, muscular and connective are etiopathogenic factors of pelvic floor pathology^18^.

In pregnancy, the entire organism is modified to allow fetus development. Changes experienced by all organic systems are increasing as pregnancy progresses and return to normal at the end of pregnancy. There is a lot of information about pathophysiology of gravity changes in cardiovascular, pulmonary, metabolic, or genital adaptations^17^. However, knowledge of changes that occur in pelvic floor’s soft, muscular, or nervous tissues is still limited.

Our study presents the influence and importance of the different obstetric background in the recurrence of urinary infections, the nulliparity, eutocic births and dystocic delivery associated with a good health state or predisposing factors to suffer RUTI.

## Objective

Investigate the relationship between the obstetric events and the occurrence of RUTIs in nulliparous and multiparous women.

## Methods

This is a retrospective multicenter observational study.

In the following centers, 588 women were treated by RUTI between January 2015 and December 2019: University Hospital of Salamanca of Salamanca (Salamanca, 37007, Spain), Hospital Virgen del Castañar of Béjar (Béjar, 37700, Salamanca, Spain), Health Center of Peñaranda (Peñaranda de Bracamonte, 37300, Spain), María Auxiliadora Health Center of Bejar (Béjar, 37700, Salamanca).

In the following centers, 126 women were treated for renal cancer, without presenting a history of UTI between January 2009 and December 2019: University Hospital of Salamanca of Salamanca (Salamanca, 37007 Spain), Hospital Virgen de la Vega of Salamanca (Salamanca, 37007, Spain), Hospital Santisima Trinidad of Salamanca (Salamanca, 37007, Spain), University Hospital of Avila (Ávila, 05004, Spain).

- Groups of study:
- Group G.RUTI (n = 294): women with RUTI.
- Group G.NON.RUTI (n = 126): women previously treated and cured of renal carcinoma without history of UTI (control group).
- Variables: age, body mass index (BMI) and gynecological and obstetric history were analized.

### Statistic analysis

It was used a statistical package IBM Corp. Released 2017. IBM SPSS Statistics for Windows, Version 25.0. Armonk, NY: IBM Corp. Results were analyzed with descriptive statistics, ANOVA analysis of variance (with Scheffe’s test for normal samples and Kruskal–Wallis for other distributions), Fisher's exact test, Pearson and Spearman correlation studies and multivariate analysis multiple regression, *p* < 0.05 was considered statistically significant.

### Ethical issues

Authors declare there are not any conflicts of interest. The authors confirm that all methods were carried out in accordance with relevant guidelines and regulations.

This study with code 2018/230/235 was approved by the Ethical Research Committees with Medicines of University Hospital of Ávila (Ávila, Spain).

Authors declare that informed consent was not applicable because it was an observational retrospective study. Ethical Research Committees with Medicines (CEIm) and the laws in the country and in the state Castilla y León approve that these types of studies do not require informed consent, only with the approval of the committee we can proceed with the study.

Costs: the financing of the study was supported by the Association for the Promotion of Training and Research in Surgical Specialties in Castilla y León (APFIEQ-CyL), Salamanca, 37007, Spain.

## Results

### Age

The mean age was 61.04 years, standard deviation (SD) 6.50, median 61, range 19–92. Age in G.RUTI (mean 56.77 y.o.; SD 4.46; range 19–85) was lower than in G.NON.RUTI (mean 71 y.o., SD 6.73, range 25–92) showed significant differences (*p* = 0.0001).

### BMI

The mean BMI was 26.95 kg/m^2^, SD 4.45, median 25.71, range 19.23–35.56. There were no differences between G.RUTI (mean 26.86, SD 4.48; 19.23–35.56) and G.NON.RUTI (mean 27.15, SD 4.38; 19.03–35.06) (*p* = 0.6118).

### Gynecological and obstetric history

**Figure 1:**
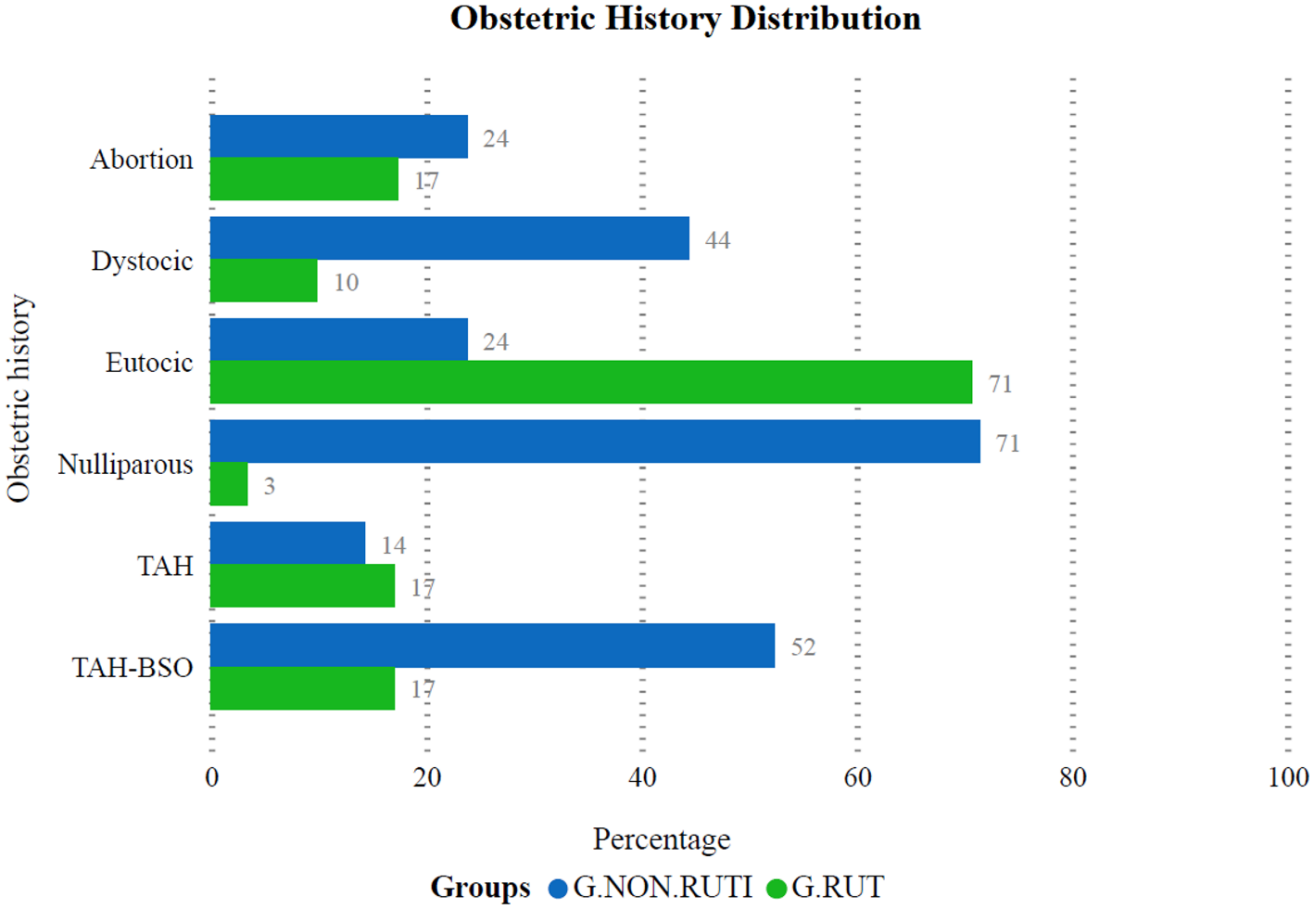
Percentages of distribution of obstetric history and conditions in women with urinary tract infections (G.RUTI) and without urinary tract infections (G.NON.RUTI). *TAH* Total abdominal hysterectomy without adnexectomy, *TAH-BSO* Hysterectomy with adnexectomy (total abdominal hysterectomy and bilateral salpingo-oophorectomy).

The comparison of the obstetric background between G.RUTI and G.NON.RUTI History of nulliparity, dystocic delivery, and hysterectomy with adnexectomy were significantly higher in the control group. Eutocic delivery history was higher in G.RUTI (*p* = 0.0001), it was not significant in history of abortion (*p* = 0.1381) and hysterectomy without adnexectomy (*p* = 0.5640) between the groups (Table 1; Fig. 1).

**Table 1:** Comparison of obstetric history and conditions between G.RUTI and G.NON.RUTI.

Obstetric history	G. RUTI (n = 294)		G.NON.RUTI (n = 126)		*P *value
	n	%	n	%	
Nulliparous	20	3.40	90	71.42	0.0001*
Eutocic	416	70.74	30	23.80	0.0001*
Dystocic	58	9.86	56	44.44	0.0001*
Abortion	102	17.34	30	23.8	0.1381
TAH	100	17.00	18	14.28	0.5640
TAH-BSO	100	17.00	66	52.38	0.0001*

G.RUTI: women with recurrent urinary tract infections. G.NON.RUTI: women without urinary tract infections.

### Multiple regression

**Figure 2:**
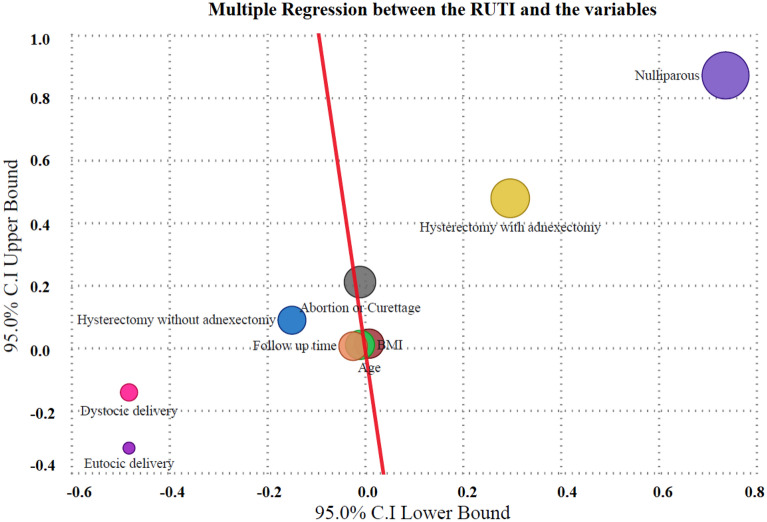
Mutiple regression between the RUTI and the variables.

A multiple regression analysis was conducted on the association of the variables with the presence or absent of recurrent urinary tract infection (R Square 0.6259; Adjusted R Square 0.6175, *p* value 0.0296, 95.0% C.I lower − 0.511 and upper 0.338), shown in Fig. 2. The direct positive association between the RUTI and the variable age, nulliparous, hysterectomy with adnexectomy was significant. Abortion or curettage was not significant (Table 2). The direct negative association between the RUTI and the variables eutocic and dystocic delivery was significant. BMI, follow up time, hysterectomy without adnexectomy was not significant (Table 2).

**Table 2:** Mutiple regression between the RUTI and the variables, positive or negative correlations of the variable on a global sample.

	Unstandardized coefficients B	Standardized coefficients Beta	*p* value	95.0% CI lower bound	95.0% CI upper bound
(Constant)	− 0.086		0.0296	− 0.511	0.338
Age	0.011	0.396	0.0004	0.009	0.013
BMI	− 0.001	− 0.006	0.888	− 0.010	0.009
Follow up time	− 0.009	− 0.064	0.193	− 0.024	0.005
Nulliparous	0.804	0.759	0.00016	0.737	0.871
Eutocic delivery	− 0.401	− 0.434	0.0009	− 0.482	− 0.321
Dystocic delivery	− 0.312	− 0.176	0.0003	− 0.482	− 0.143
Hysterectomy without adnexectomy	− 0.030	− 0.025	0.618	− 0.149	0.088
Hysterectomy with adnexectomy	0.388	0.384	0.0005	0.297	0.478
Abortion or Curettage	0.100	0.088	0.075	− 0.010	0.210

## Discussion

UTI are a major health problem in women: they affect more than 50% at some point in life and 25% become RUTI. Despite investigating RUTI causes, relating them to estrogenic status and pelvic floor function, relationship between obstetric history and RUTI occurrence is unknown. Pregnancy and childbirth impact on perineal muscle deterioration leading to urinary incontinence and pelvic prolapse is well established.

Regarding obstetric and gynecological history distribution, we found that women without RUTI (G.NON.RUTI), were more nulliparous, 71.42% compared to 3.40% of women with RUTI (G.RUTI), we found a positive and direct correlation between RUTI and the major presence of nulliparous background, in others studies^19^^,^^20^, exposed the presence pregnancies and deliveries influences when presenting pelvic floor dysfunctions, such as UTI.

However, eutocic delivery history is much more frequent (70.74%) in women with RUTI (G.RUTI) than in those with non RUTI (23.80%), we found a negative correlation. Dystocic deliveries, curiously, are more frequent in women without RUTI 44.44%, other publications conclude that dystocic deliveries are related to pelvic floor dysfunction while in our study, we observed there is a negative correlation wich could indicated dystocic delivery is related to the less presence of RUTI^19^^,^^21^.

In data provided by our study, we found no difference in abortion history distribution between women with RUTI and those with kidney cancer, there was a positive and direct correlation (*p* = 0.075). There is also no difference in distribution of history of hysterectomy without adnexectomy, there was a positive and direct correlation (*p* = 0.618).

A truly relevant fact is that history of hysterectomy with adnexectomy is more frequent in the control group without RUTI (17.00%) and with a positive and direct correlation (*p* = 0.0005) which could indicated is related to the major presence of RUTI. This is particularly, given that both hysterectomy with adnexectomy and dystocic deliveries are two conditions that are usually associated with development of RUTI.

In reference to hysterectomy with adnexectomy, adnexectomy involves an artificial menopause, and estrogen deficiency is assumed to lead to vaginal mucosa atrophy that could lead to RUTI. Dystocic deliveries have been found to be generally associated with poorer functionality of the general pelvic floor^19^^,^^22^, so data obtained is surprising compared to previous literature.

### Study limitations

First difficulty that we encountered in this work is that there are hardly any publications that relate the obstetric history with the occurrence of RUTI.

Second, we found it difficult to find a control group; at first, we considered that control group were kidney transplant women, but they had many pathological urinary tract antecedents and were not useful as a control group. For this reason, we chose women treated only for kidney cancer and cured of it.

Third difficulty was that, when comparing age, women with kidney cancer were older, both median age and mean age. However, range is highly overlapping between the two groups, because range of women investigated by RUTI was 19–85 and that of women with kidney cancer was 25–92. Therefore, we decided to continue with this comparative control group.

## Conclusions

Women with recurrent urinary tract infections are related with more frequent history of eutocic delivery (70.74%) compared to those without infections (23.80%). However, nulliparity (3.4% vs. 71.42%), dystocic delivery (9.86% vs. 44.44%) and hysterectomy with adnexectomy (17% vs. 52.38%) are more frequent in women without recurrent urinary tract infections. History of abortion (17.34% vs. 23.8%) and hysterectomy without adnexectomy (17% vs. 52.38%) are not related to having or not having a UTI.

The most prevalent and related obstetric antecedent in women with RUTI is eutocic delivery associated with good evolution in the presence of recurrent urinary tract infections.

There is a relation between women with more deteriorated health state with RUTI and the dystocic delivery antecedent, with the condition of concomitant urinary incontinence being more prevalent.
